# Implication of the IL-10-Expression Signature in the Pathogenicity of *Leptospira*-Infected Macrophages

**DOI:** 10.1128/spectrum.02595-21

**Published:** 2022-05-31

**Authors:** Li-Fang Chou, Ting-Wen Chen, Huang-Yu Yang, Ya-Chung Tian, Ming-Yang Chang, Cheng-Chieh Hung, Chih-Ho Lai, Shen-Hsing Hsu, Chung-Ying Tsai, Yi-Ching Ko, Jang-Hau Lian, Chih-Wei Yang

**Affiliations:** a Kidney Research Center, Chang Gung Memorial Hospital, Linkou, Taiwan; b Institute of Bioinformatics and Systems Biology, National Yang Ming Chiao Tung University, Hsinchu, Taiwan; c Department of Biological Science and Technology, National Yang Ming Chiao Tung University, Hsinchu, Taiwan; d Center for Intelligent Drug Systems and Smart Bio-devices (IDS^2^B), National Yang Ming Chiao Tung University, Hsinchu, Taiwan; e Department of Nephrology, Chang Gung Memorial Hospital, Linkou, Taiwan; f College of Medicine, Chang Gung University, Taoyuan, Taiwan; g Department of Microbiology and Immunology, Chang Gung University, Taoyuan, Taiwan; h Molecular Infectious Disease Research Center, Chang Gung Memorial Hospital, Taoyuan, Taiwan; i Genomic Medicine Core Laboratory, Chang Gung Memorial Hospital, Linkou, Taiwan; University of North Dakota

**Keywords:** bone marrow-derived macrophage, IL-10, *Leptospira* spp., macrophage activation

## Abstract

Leptospirosis, an emerging infectious disease caused by pathogenic *Leptospira* spp., occurs in ecoregions with heavy rainfall and has public health implications. Macrophages are the major anti-*Leptospira* phagocytes that infiltrate the kidneys during renal leptospirosis, which is caused by leptospires residing in the renal tubules. The pathogenicity of *Leptospira* spp. in immune effector cells such as macrophages is not well understood. To evaluate this pathogenesis, we characterized and compared the transcriptome-wide alterations in macrophages infected with pathogenic and nonpathogenic *Leptospira* spp. Using transcriptome data and quantitative reverse transcription PCR analysis, at 2 h postinfection, the hypoxia-inducible factor-1α-dependent glycolysis pathway was implicated in pathogenic *Leptospira*-infected macrophages but not in nonpathogenic leptospiral infections. Immune-related biological processes were mostly activated in pathogenic *Leptospira*-infected macrophages, and flow cytometry investigations revealed that classically activated macrophages represent the predominant polarization status. At 24 h after infection, biological pathways associated with interleukin-10, IL-10, signaling the induction of macrophage tolerance, as well as higher levels of IL-10 mRNA and protein expression, were observed in nonpathogenic *Leptospira*-infected macrophages compared to in pathogenic leptospiral infection. Following leptospiral infection of macrophages, strong IL-10-expressing transcriptome signatures were observed following nonpathogenic leptospiral infection. The transcriptional programs generated in *Leptospira*-infected macrophages revealed an inflammatory milieu following the production of a critical anti-inflammatory cytokine, IL-10, which is implicated in controlling the pathogenicity of activated macrophages. These findings imply that IL-10-mediated anti-inflammatory responses and tolerance in activated macrophages induced by nonpathogenic *Leptospira* spp. infection reduce inflammation and tissue damage, thus providing a potential therapeutic target for leptospirosis.

**IMPORTANCE** Activation of macrophages by *Leptospira* spp. infection is thought to be involved in the pathogenesis of leptospirosis. To evaluate the innate macrophage responses to *Leptospira* spp., specifically pathogenic versus nonpathogenic *Leptospira* spp., we characterized the entire transcriptome-wide alterations in infected macrophages. We showed that hypoxia-inducible factor-1α and immune-related pathways are activated in pathogenic leptospiral-infected macrophages. We confirmed the significantly high levels of IL-10-expressing signatures and tolerance in activated macrophages caused by nonpathogenic *Leptospira* infection. Furthermore, nonpathogenic leptospiral infections attenuated macrophage activation responses. These findings suggest a potential therapeutic strategy for the immune microenvironment caused by macrophage activation driven by IL-10 overexpression, which may contribute to regulating inflammation in leptospirosis.

## INTRODUCTION

Leptospirosis, an emerging infectious zoonotic and waterborne disease caused by pathogenic *Leptospira* spp., is endemic to tropical/subtropical ecoregions with heavy rainfall and has public health implications (1). Because its incidence is increasing globally, leptospirosis is a growing public health issue. Leptospirosis causes an estimated more than 1 million cases and 60,000 deaths annually worldwide (1). The clinical manifestations of human leptospirosis vary from asymptomatic infection to severe diseases with multiorgan damage, including hepatic dysfunction and renal failure, all of which are associated with immune manifestations (2).

Leptospiral infections in humans are caused by direct or indirect contact with infected reservoir animals, which carry the pathogen in their renal tubules/urine, leading to spread throughout the ecosystem (3). *Leptospira* species invade the human body through skin abrasions or mucosal surfaces, enter the bloodstream, and disseminate to various organs where they cause tissue damage (2). Renal lesions are associated with leptospirosis in humans.

Asymptomatic leptospiral chronic infections in the kidneys can progress to chronic tubulointerstitial nephritis and renal fibrosis based on our previous studies (4–6). Recent studies suggested that *Leptospira* spp. evade the innate immune response of the host through its resistance to the complement system and interactions of leptospiral components with host pattern recognition receptors (PRRs), such as Toll-like receptors (TLRs) and the nucleotide-binding oligomerization domain (NOD)-like receptor (NLR) family (7).

The cellular innate immune system, including neutrophils and macrophages, is the first line of defense against infection. This system is involved in early recognition and elimination of invading pathogens. Macrophages are dynamic and heterogeneous cells that directly defend against bacteria within cells and secrete cytokines to activate immune responses. Surface receptors on macrophages, such as TLRs and complement receptors, and assist the host in recognizing pathogens and presenting antigens for adaptive immunity (8). During spirochete infections, macrophages are essential for controlling the bacterial burden and tissue inflammation. Studies on the function of macrophages in leptospiral infection indicated that macrophages can effectively phagocytize leptospires (9–11). Pathogenic Leptospira interrogans, but not nonpathogenic Leptospira biflexa, can survive, replicate, and be released from cells to the extracellular milieu in infected murine macrophages at 24 h after infection (9). L. interrogans, an uncommon intracellular pathogen, may block NOD1/2 recognition through peptidoglycan modification induced by leptospiral lipoprotein LipL21 binding, thereby escaping from the phagolysosome to the cytosol of macrophages (7). Differentially regulated genes in L. interrogans-infected murine and human primary macrophages are involved in biological signaling pathways related to antigen presentation, membrane regulation, TLR signaling pathway, innate immune responses, and cytokine and chemokine secretion (10).

Macrophages are the main infiltrating anti-*Leptospira* phagocytes in the kidneys of infected mice (12). According to previous *in vivo* studies, tissue macrophages are derived from circulating bone marrow-derived monocytes (13). A recent study reported that a renal biopsy from severe leptospirosis associated with oliguric renal failure showed inflammatory infiltration in the tubules/interstitium and accumulation of macrophages, predominantly the classical M1 phenotype (14). Macrophage activation and polarization are dynamic processes controlled by local microenvironmental signals (15). Polarized macrophages have been broadly classified into two types based on their activation state and roles: classically activated M1 (proinflammatory profile; higher levels of CD80 and nitric oxide synthase expression used as phenotypic markers) and alternatively activated M2 (anti-inflammatory profile; express CD206 and arginase I as markers) macrophages (16). In response to various signals, macrophages can differentiate from monocytes *in vitro* and may undergo classical M1 activation (stimulated by TLR ligands such as bacterial lipopolysaccharide (LPS), and type I inflammatory cytokines) or alternative M2 activation (stimulated by interleukin (IL)-4 or IL-13) (17). Classically activated M1 macrophages secrete proinflammatory cytokines and mediate tissue injury, whereas alternatively activated M2 macrophages produce arginase I and anti-inflammatory cytokines such as IL-10, which reduce inflammation and contribute to tissue repair (17–19). Moreover, LPS-tolerant macrophages are polarized toward the M2 phenotype (20).

IL-10 is an anti-inflammatory cytokine and potent immunoregulator during infection (21). Macrophages are key sources of IL-10 during infection. Several studies have reported that IL-10 protects the infected host by reducing pathogen-induced immunological disorders or by hijacking the IL-10-related pathway to subvert the host immune response (21, 22). Recent studies showed that LPS-tolerant monocytes/macrophages express low levels of M1-associated genes and high levels of M2-associated genes such as IL-10 (20). IL-10 produced by macrophages prevents adjacent cells from becoming classically activated M1 macrophages, allowing cells to self-regulate (23). IL-10 plays a role in limiting the host immune response to pathogens and in maintaining tissue homeostasis by reducing the immunological response of the host to infection, thereby preventing damage to the host.

The leptospire–macrophage interaction is a common model used to examine the initial response to leptospiral infection. Transcriptomic studies of leptospiral infections have been conducted using *in vitro* macrophage infection models. Based on the global responses of pathogenic L. interrogans to macrophage-derived cells, as shown by Xue et al., modulation of leptospiral outer membrane proteins may be an immune evasion strategy used by pathogenic L. interrogans to avoid host innate immunity during the early stage of infection (24). Macrophage responses after leptospiral infection have been described by Araujo et al. (25, 26). Microarray-based gene expression profiles showed that at 6 h after infection with different strains of *Leptospira* spp. in the murine macrophage cell line J774A.1, apoptosis and cell repair/movement/death/survival were significantly activated following infection with the virulent strain (26).

In this study, we performed global analysis of gene expression associated with murine bone marrow-derived macrophages (BMDMs) induced by leptospiral infection using *ex vivo* infection models. To better understand how macrophages alter their gene expression in response to pathogenic and nonpathogenic *Leptospira* spp. infection, we performed high-throughput next-generation sequencing (NGS) for comprehensive transcriptomic analysis to explore the contribution of biological molecules and signaling pathways in infected macrophages. Our results provide a foundation for further investigation of the pathogenesis of leptospirosis. Together, these findings will facilitate further molecular studies of the innate immune response to leptospiral infections. A better understanding of the molecular pathogenesis of persistent intracellular leptospire-derived infected macrophages may lead to improved diagnostics, therapeutics, and prophylactics for leptospirosis.

## RESULTS

### Macrophage morphology changes following leptospiral infection.

**FIG 1:**
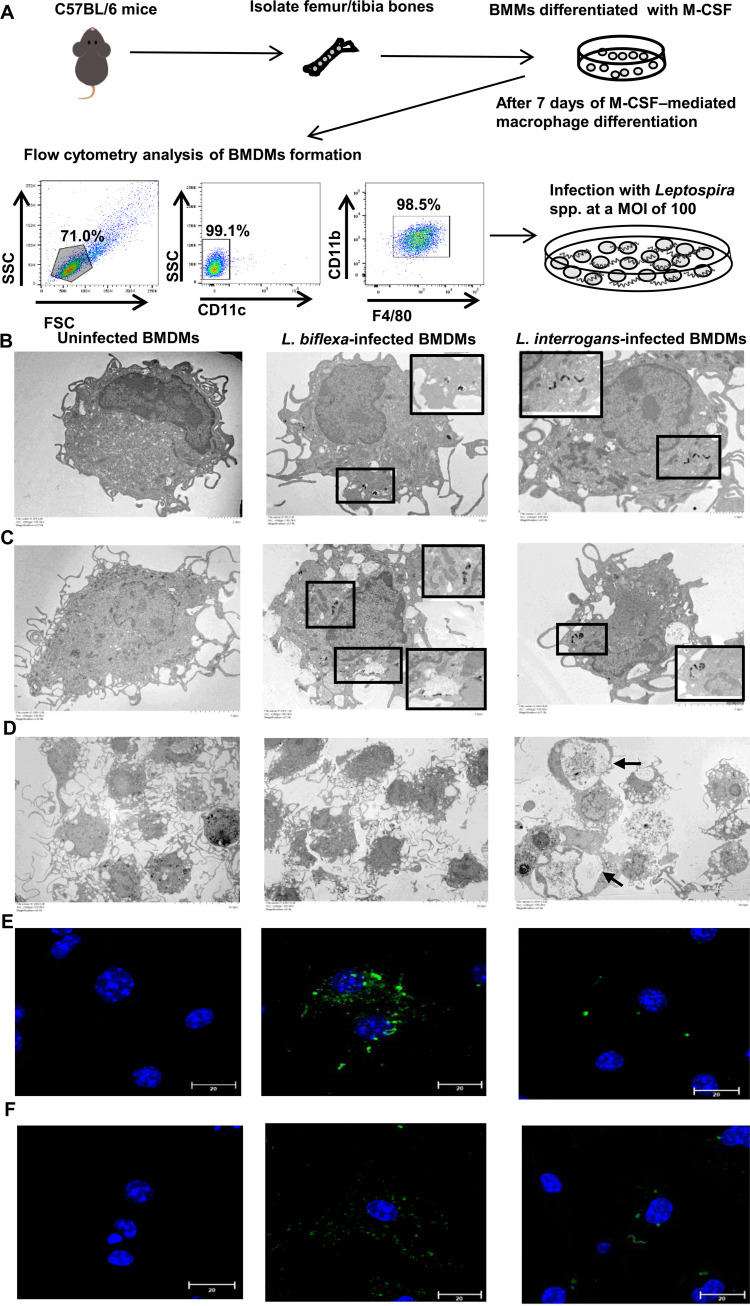
Differential morphology of cells and leptospires uptake in BMDMs infected with *Leptospira* spp. (A) Schematic representation of an *ex vivo* model of murine BMDMs infected with *Leptospira* spp. Mouse bone marrow suspensions were cultivated in medium containing macrophage colony-stimulating factor for 7 days; adherent cells were harvested for assays using flow cytometry. The purity of F4/80^+^/CD11b^+^/CD11c- BMDMs was approximately 90%, and precise numbers of macrophages were plated for experimental infection with *Leptospira* spp. The cellular architecture of *Leptospira*-infected BMDMs at 2 h (B) and 24 h (C and D) postinfection, as visualized using transmission electron microscopy. Scale bar, 2.0 μm (B); 5.0 μm (C); and 20.0 μm (D). Intracellular leptospiral localization patterns in infected macrophages are depicted in an interior frame; these areas are magnified in the corner of the photos. Immunofluorescence detection of intracellular *Leptospira* spp. in infected BMDMs at 2 h (E) and 24 h (F) postinfection. BMDMs were stained with anti-Lipl32 antibody for L. interrogans (green), anti-*L. biflexa* antibody to *L. biflexa* (green), and DAPI (blue) to visualize the cell nucleus. Uninfected BMDMs were analyzed as negative controls (2 and 24 h). Samples were analyzed using confocal microscopy (scale bar, 20.0 μm; magnification 1,000×). BMDM, bone marrow-derived macrophage.

To investigate the role of activated macrophages after infection with pathogenic and nonpathogenic *Leptospira* spp., we used murine bone marrow cells grown in the presence of recombinant macrophage colony-stimulating factor, a growth factor that causes myeloid bone marrow progenitor cells to differentiate into BMDMs (27). On day 7 of culture, over 90% of cells were negative for the dendritic cell marker CD11c but positive for the macrophage markers, F4/80 and CD11b, demonstrating that the cells were pure and had matured into macrophages (Fig. 1A).

Cell morphological differences and uptake of leptospires in BMDMs were evaluated using transmission electron microscopy (TEM). As shown in Fig. 1B and Fig. S1A in the supplemental material, in pathogenic *Leptospira*-infected macrophages, mitochondria with a characteristic electron-dense matrix predominated in the early stage of infection (2 h postinfection) compared to mitochondria with a loss of electron density in uninfected and nonpathogenic *Leptospira*-infected macrophages, indicating increased mitochondrial activity in pathogenic *Leptospira*-infected macrophages. In addition to the difference in mitochondria, intact L. interrogans was localized in the cytosol of BMDMs, and degraded *L. biflexa* was found within vacuoles at 2 and 24 h postinfection (area delineated by a rectangle in Fig. 1B–C and Fig. S1B).

As shown in Fig. 1B–C, the amount of *L. biflexa* in infected BMDMs was greater than that of L. interrogans in BMDMs in a single cell; the distribution of intracellular fluorescent *Leptospira* spp. was higher in infected BMDMs at 2 and 24 h after infection (Fig. 1E–F and Fig. S1C). These findings indicate that intact L. interrogans were present in macrophages at 2 and 24 h after infection, whereas *L. biflexa* was degraded (Fig. S1B). TEM images of BMDMs infected with L. interrogans at 24 h postinfection revealed abnormal compartments, including a megasome-like structure surrounded by the membranes of BMDMs (Fig. 1D, arrow). At 24 h after infection, morphological changes in BMDMs infected with L. interrogans were compared with those in uninfected and *L. biflexa*-infected macrophages (Fig. 1D). As shown in Fig. S1D, the percentage of early apoptotic cells in *L. biflexa*-infected BMDMs was higher than that in L. interrogans-infected BMDMs at 24 h after infection, suggesting that the presence of large vacuoles in L. interrogans-infected BMDMs is unrelated to apoptotic cell death. The results suggest that the proliferation of infected cells resulted in a decreased percentage of late apoptotic cells after a 24-h infection compared to 2-h infection.

### Distinct transcriptional signatures of BMDMs infected with pathogenic and nonpathogenic *Leptospira* spp.

**FIG 2:**
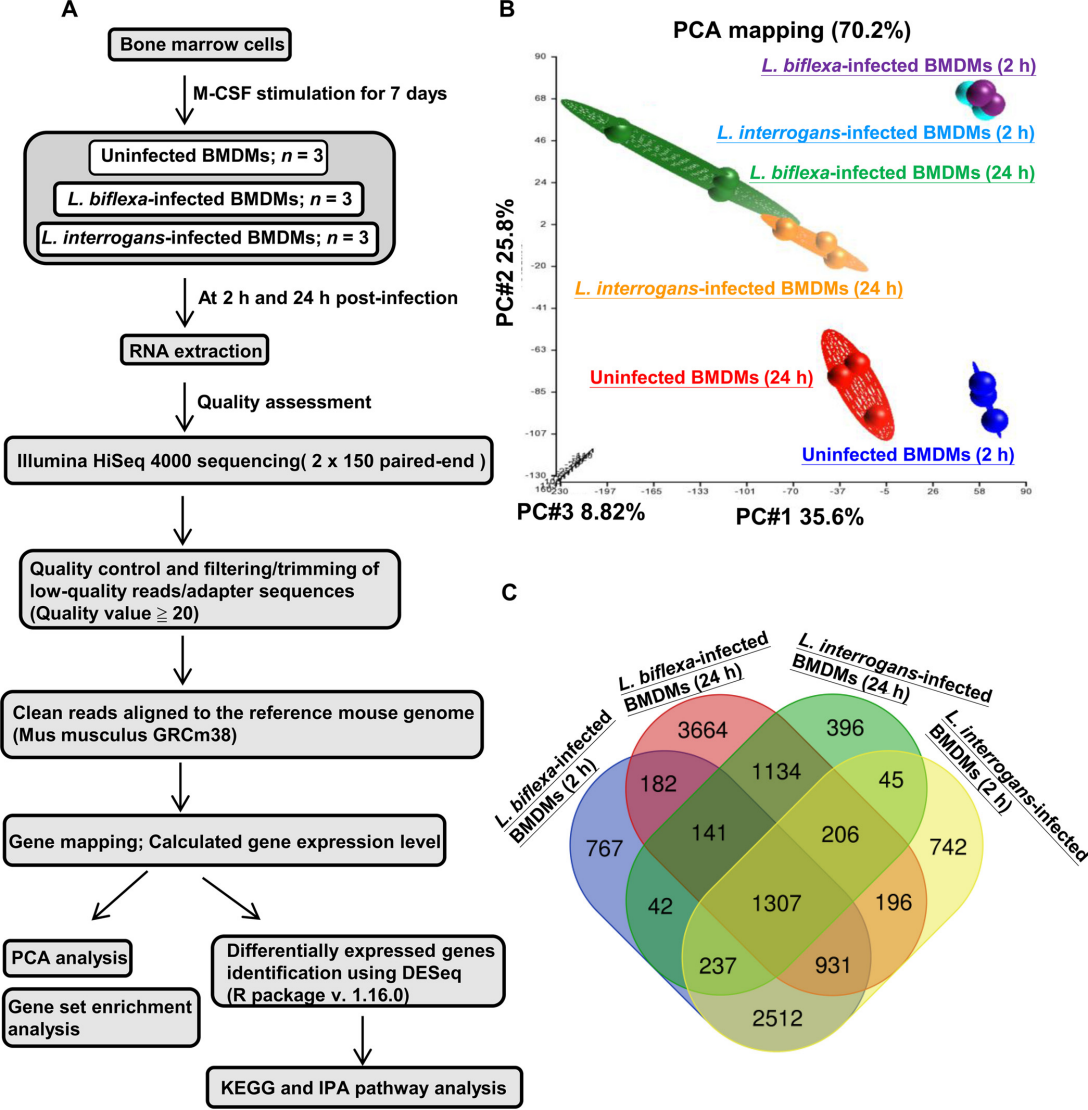
Changes in global macrophage transcriptomes in response to leptospiral infection. (A) Schematic representation of transcriptional profiles of infected macrophages. (B) Global BMDM transcriptome profiles determined using PCA. (C) Venn diagram comparison showing common and unique genes differentially expressed in infected macrophages at 2 and 24 h after infection. BMDM, bone marrow-derived macrophage; PCA, principal coordinate analysis.

Illumina RNA-sequencing (RNA-seq) was performed for transcriptomics profiling in murine BMDMs infected with pathogenic L. interrogans and nonpathogenic *L. biflexa* for 2 and 24 h, respectively, to examine the direct effects of *Leptospira* spp. on macrophages (Fig. 2A). Next-generation sequencing was performed using a paired-end 2 × 150 bp library on the HiSeq4000 platform (Illumina, San Diego, CA, USA). Table S1 summarizes the sequencing data for each sample. On average, we obtained 60 million filtered and cleaned 130-nucleotide reads mapped to mouse genomes. To evaluate global changes in the BMDM transcriptome in response to leptospiral infection, principal-component analysis (PCA) was performed on three independent biological replicates for each infection time point and for each group to visualize the relationships between the tested samples. The PCA plot of the RNA-seq data showed that the transcriptomic profiles were clustered by different experimental samples and infection periods. Segregation of uninfected and infected groups is shown in Fig. 2B and indicates changes in gene expression profiles following infection. Samples at two time points (2 and 24 h) were clustered independently. At 2 h postinfection, samples infected with pathogenic and nonpathogenic *Leptospira* spp. clustered together (Fig. 2B). Our results indicate that the three-group data (uninfected, L. interrogans-infected, and *L. biflexa*-infected BMDMs) were separated in a time-dependent manner, and the gene expression profiles of BMDMs infected with L. interrogans were distinct from those of *L. biflexa* at 24 h postinfection.

At 2 h postinfection, we identified 6,176 differentially expressed genes (DEGs) between BMDMs infected with L. interrogans and uninfected BMDMs. Of these, 27.15% of genes were upregulated, and 72.85% were downregulated. There were 3,508 DEGs in BMDMs infected with L. interrogans compared to in uninfected BMDMs after 24 h of infection, with 36.52% upregulated and 63.48% downregulated (Table 1). In BMDMs infected with *L. biflexa*, at 2 and 24 h postinfection, 6,119 (26.83% upregulated and 73.17% downregulated) and 7,761 (47.84% upregulated and 52.16% downregulated) genes were differentially expressed, respectively (Table 1). The extensive downregulation of gene expression in infected BMDMs suggests that biological pathways were slowed during leptospiral infection. Fig. 2C shows that among the four groups analyzed, the largest number of differentially expressed genes (3,664 DEGs; 1,145 gene transcripts were downregulated, and 2,519 gene transcripts were upregulated) was observed at 24 h after infecting BMDMs with *L. biflexa*, showing the dramatic variation in the transcriptome profiles of the *L. biflexa*-infected BMDM groups (at 24 h postinfection).

**TABLE 1:** Number of DEGs and significantly enriched pathways in bone marrow-derived macrophages following leptospiral infection

Groups	Infection times^*a*^	DEGs^*b*^			KEGG pathways^*c*^	Ingenuity canonical pathways^*c*^
		Up-regulated genes (%)	Down-regulated genes (%)	Total		
*L. biflexa*	2	1,642 (26.83)	4,477 (73.17)	6,119	14	36
	24	3,713 (47.84)	4,048 (52.16)	7,761	8	24
L. interrogans	2	1,677 (27.15)	4,499 (72.85)	6,176	14	29
	24	1,281 (36.52)	2,227 (63.48)	3,508	24	61

a Infection times, hours.

b Numbers of DEGs (the absolute value of FC ≥ 2; *P* value <  0.05) based on RNA-seq data. The DEGs in comparisons of *L. biflexa*-infected BMDMs *vs.* uninfected BMDMs and L. interrogans-infected BMDMs *vs* uninfected BMDMs.

c The *P* value <1E-5 was used as a threshold to select significant pathways enriched at KEGG using clusterProfiler and canonical pathways determined from ingenuity pathway analysis.

**FIG 3:**
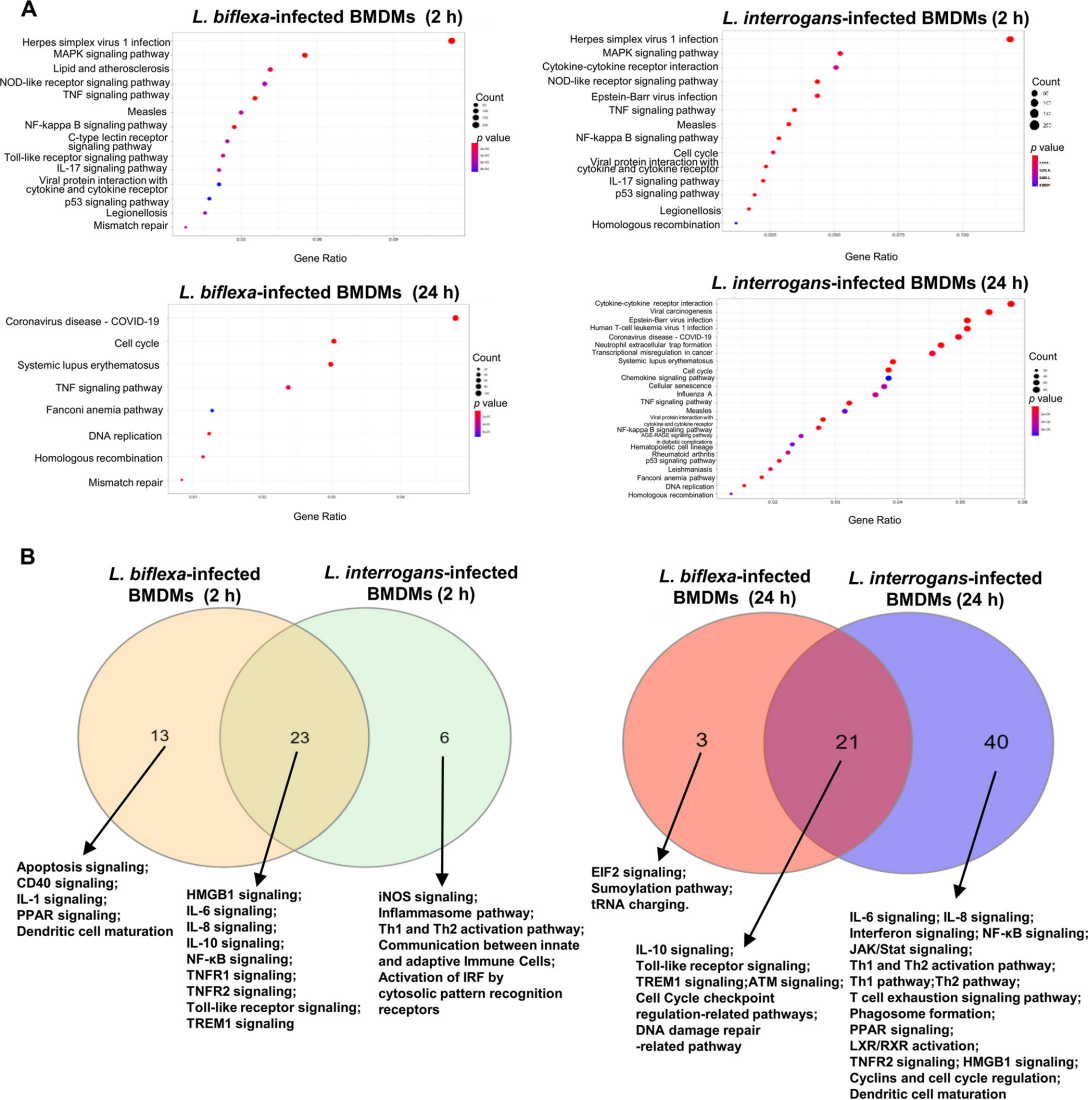
Enrichment of functional pathways identified from global transcriptional profiles of infected macrophages. (A) Dot plots showing significantly enriched KEGG pathways (*P < *1E-5) in infected macrophages. The gene ratio on the *x* axis is the relative abundance of genes in each KEGG term, and the *y* axis shows the KEGG terms. Dot size represents the number of differentially expressed genes in each term, and the circle color of each dot represents the *P* value of each term. (B) Significantly enriched IPA canonical pathways (*P < *1E-5) identified in macrophages infected with Leptospira biflexa or Leptospira interrogans after infection at 2 and 24 h. Venn diagram showing overlapping significantly enriched canonical pathways for comparison. IPA, ingenuity pathway analysis; KEGG, Kyoto Encyclopedia of Genes and Genomes.

To examine the molecular mechanisms underlying *L. interorgan*- and *L. biflexa-*infected BMDMs, the identified DEGs were investigated using the Kyoto Encyclopedia of Genes and Genomes (KEGG) pathway enrichment analysis and the Ingenuity Pathway Analysis (IPA) with the filtering option *P < *1E-5. Table 1 lists the number of significantly enriched canonical pathways. KEGG pathway enrichment analysis showed that at 2 and 24 h postinfection, pathogenic *Leptospira*-infected BMDMs had 14 and 24 significantly enriched pathways, respectively, and nonpathogenic *Leptospira*-infected BMDMs had 14 and 8 significantly enriched pathways, respectively. At 2 h postinfection, the DEGs were significantly enriched in the “IL-17 signaling pathway,” “nuclear factor (NF)-kappa B signaling pathway,” and “tumor necrosis factor (TNF) signaling pathway” in both L. interrogans- and *L. biflexa*-infected BMDMs (Fig. 3A). In nonpathogenic *Leptospira*-infected BMDMs, pathways related to PRRs, including the “C-type lectin receptor signaling pathway,” “NOD-like receptor signaling pathway,” and “Toll-like receptor signaling pathway” were significant; however, pathogenic *Leptospira*-infected BMDMs were enriched only in the “NOD-like receptor signaling pathway.”

In addition, the “cytokine-cytokine receptor interaction” pathway was enriched in BMDMs infected with pathogenic *Leptospira* spp. but not in those infected with nonpathogenic *Leptospira* spp. KEGG pathway enrichment analysis revealed significant enrichment of “cell cycle,” “DNA replication,” and “homologous recombination”-associated pathways for both L. interrogans- and *L. biflexa*-infected BMDMs at 24 h postinfection. Pathways associated with the “chemokine signaling pathway,” “cytokine-cytokine receptor interaction,” “NF-κB signaling pathway,” and “neutrophil extracellular trap formation” were enriched in L. interrogans-infected BMDMs but not in *L. biflexa*-infected BMDMs (Fig. 3A).

The results of IPA enrichment analysis of the DEGs are shown in Fig. 3B. Both L. interrogans- and *L. biflexa*-infected BMDMs infected for 2 and 24 h were enriched in “Toll-like receptor signaling,” “triggering receptor expressed on myeloid cells 1 (TREM1) signaling,” and “interleukin 10 signaling.” Using a Z-score of >2 as the threshold for significant activation, “TREM1 signaling,” “role of PRRs in recognition of bacteria and viruses,” “IL-6 signaling,” and “high mobility group box 1 signaling” were significantly active in *L. biflexa*- and L. interrogans-infected BMDMs, respectively, at 2 h postinfection (Table S2). Furthermore, immune-related biological processes, such as “IL-6 signaling” (Z-score = 2.655), “NF-κB signaling” (Z-score = 2.188), “T cell exhaustion signaling pathway” (Z-score = 2.466), “Th1 pathway” (Z-score = 2.058), “Th2 pathway” (Z-score = 2.121), and “interferon signaling” (Z-score = 3.207), were significantly activated in L. interrogans-infected BMDMs at 24 h postinfection.

### High expression levels of IL-10 in macrophages infected by nonpathogenic *Leptospira* spp.

**FIG 4:**
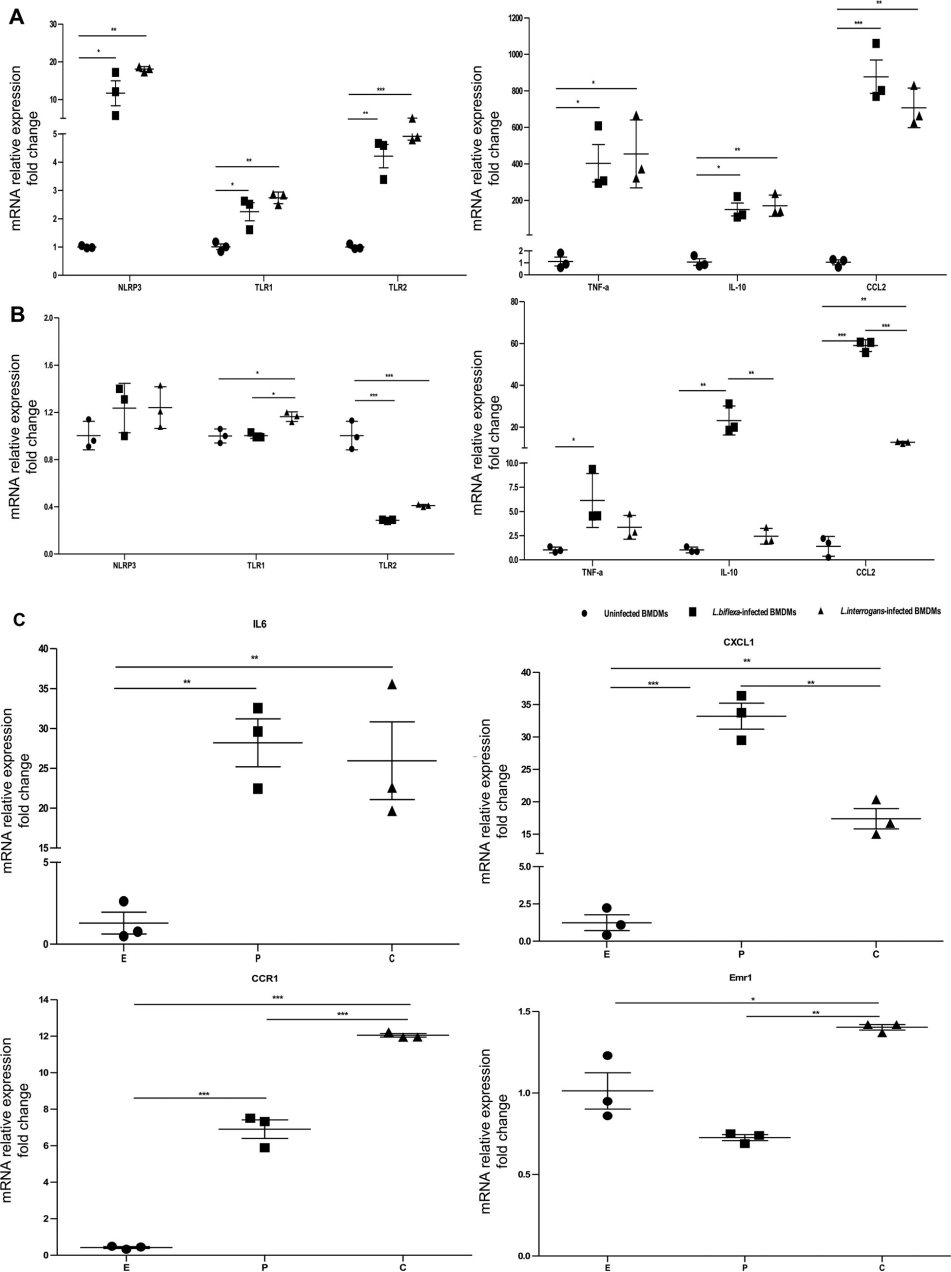
Genes differentially expressed in macrophages infected with *L. biflexa* or L. interrogans. After infection with leptospires for 2 h (A) and 24 h (B and C), relative mRNA expression levels for the indicated gene were analyzed. NLR family, pyrin domain containing 3: NLRP3; Toll-like receptor 1: TLR1; Toll-like receptor 2: TLR2; tumor necrosis factor-alpha: TNF-α; interleukin 10: IL-10; chemokine (C-C motif) ligand 2: CCL2; interleukin 6: IL-6; chemokine (C-X-C motif) ligand 1: CXCL1; chemokine (C-C motif) receptor 1: CCR1; EGF-like module containing, mucin-like, hormone: Emr1. The expression of target genes was normalized to that of GAPDH. Control condition refers to uninfected BMDM groups. Data were analyzed using a two-way analysis of variance followed by multiple comparisons tests (GraphPad Prism). Significant differences are representative of the results from multiple experimental replicates (*n *= 3). *, *P < *0.05; **, *P < *0.01; ***, *P < *0.005. E: uninfected BMDMs; P: *L. biflexa*-infected BMDMs; C: L. interrogans-infected BMDMs. BMDM, bone marrow-derive macrophage.

Based on the above results, we selected six gene transcripts involved in the TLR, NLR, and TNF pathways and proinflammatory/anti-inflammatory cytokines to validate the gene expression profiles obtained in RNA-seq analysis (Table S3). The results of quantitative real-time reverse transcription-PCR (RT-PCR) analysis of the selected genes in infected and uninfected BMDMs performed at two time points agreed with the RNA-seq transcriptomic data, indicating the reliability of the DEGs obtained from transcriptome sequencing (Fig. 4A and B). The expression of TLR1, TLR2, NLR family pyrin domain containing 3 (NLRP3), and inflammatory genes was significantly upregulated in BMDMs after leptospiral infection at early time points (2 h postinfection) (Fig. 4A). The results showed increased expression of IL-10 (9.81-fold, *P < *0.01) and chemokine (C-C motif) ligand 2 (CCL2; 4.65-fold, *P < *0.005) in *L. biflexa*-infected BMDMs compared to that in L. interrogans-infected BMDMs (Fig. 4B).

We further explored the effect of biological molecules or cytokines in the microenvironment with high expression of IL-10, which is induced by leptospiral infection, using transcriptome-wide analysis because of the high expression levels of IL-10 in macrophages infected with nonpathogenic *Leptospira* spp. By calculating the ratio (fold change ratio in L. interrogans-infected compared to in *L. biflexa*-infected BMDMs) for each expressed gene, we predicted the biological molecules or cytokines related to pathogenic *Leptospira*-infected BMDMs with low IL-10 expression or nonpathogenic *Leptospira*-infected BMDMs with high IL-10 expression. As shown in Table S4, there were 411 *L. biflexa*-induced gene transcripts in BMDMs at 24-h of infection, as demonstrated by a reduced ratio (fold change ratio in L. interrogans-infected compared to in *L. biflexa*-infected BMDMs) of <1.0, whereas 1,065 L. interrogans-induced gene transcripts showed a fold change ratio of >1.0 (Table S5). According to our RNA-seq analysis and quantitative RT-PCR validation results, the mRNA levels of C-X-C motif chemokine ligand 1 (CXCL1) were significantly higher in BMDMs infected with *L. biflexa* than in those infected with L. interrogans (*P < *0.01) (Fig. 4C). The levels of CCR1 and Emr1 mRNA expression in L. interrogans-infected BMDMs were significantly higher than those in *L. biflexa*-infected cells (Fig. 4C and Table S3). Based on these findings, the number of highly expressed gene transcripts such as C-X-C motif chemokine ligand 10 (CXCL10) and C-X-C motif chemokine ligand 9 (CXCL9) in L. interrogans-infected BMDMs with low IL-10 expression was 2.5-fold higher than that in *L. biflexa*-infected cells with high IL-10 expression, suggesting anti-inflammatory responses in BMDMs induced by nonpathogenic *Leptospira* spp. infection.

### Pathway-based gene signatures associated with leptospiral infection-induced macrophage activation.

**FIG 5:**
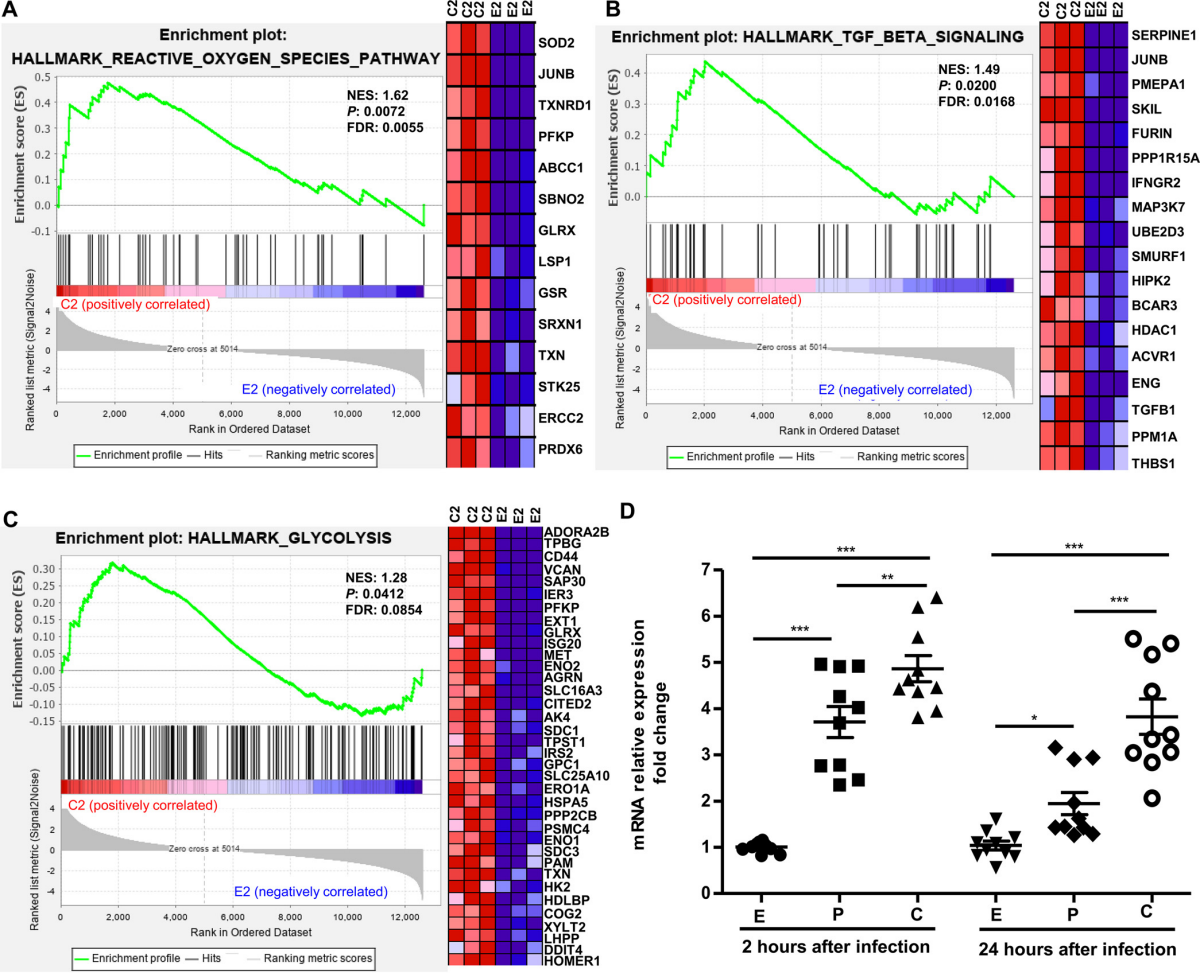
Macrophage responses at 2 h after infection with pathogenic *Leptospira* spp. Plots obtained from gene set enrichment analysis of (A) “HALLMARK_REACTIVE_OXYGEN_ SPECIES_PATHWAY,” (B) “HALLMARK_TGF_BETA_SIGNALING,” and (C) “HALLMARK_GLYCOLYSIS” are shown in L. interrogans-infected BMDMs *versus* uninfected BMDM groups but not in *L. biflexa*-infected BMDMs *versus* uninfected BMDM groups (NES > 1, *P < *0.05, and FDR < 0.25). The green curve refers to the calculation of the enrichment score. BMDM, bone marrow-derived macrophage; GSEA, gene set enrichment analysis; NES, normalized enrichment score; FDR, false discovery rate. Left panels show GSEA enrichment plots (score curves), and the heatmap on the right side of each panel is a visualization of the core enrichment genes (positively enriched associated molecules) contributing the most to the enriched pathway. Increased expression (red) and decreased expression (blue). C2, L. interrogans-infected BMDMs at 2 h postinfection; E2, uninfected BMDMs at 2 h. (D) Significant upregulation of HIF-1α mRNA levels were examined in L. interrogans-infected BMDMs compared to in uninfected BMDMs and *L. biflexa*-infected BMDMs at 2 h postinfection. HIF-1α, hypoxia inducible factor 1, alpha subunit. The expression of target genes was normalized against GAPDH. Control condition refers to uninfected BMDM groups. The data were analyzed using two-way analysis of variance followed by multiple comparisons tests (GraphPad Prism). Significant differences are representative of the results from multiple experimental replicates (*n *= 10). *, *P < *0.05; **, *P < *0.01; ***, *P < *0.005 E: uninfected BMDMs; P: *L. biflexa*-infected BMDMs; C: L. interrogans-infected.

To identify the cellular biological mechanisms underlying the regulatory pathways induced by pathogenic and nonpathogenic *Leptospira* spp. infection, gene set enrichment analysis (GSEA) was performed using RNA-seq transcriptome data. The cutoff value for the enrichment magnitude and statistical significance was defined as an absolute normalized enrichment score (NES) ≥1, a nominal *P* value of <0.05, and a false discovery rate (FDR) q-value of <0.25. Cellular pathways associated with leptospiral infection were significantly enriched in *Leptospira*-infected BMDMs according to GSEA performed with hallmark gene sets collected from the Molecular Signatures Database (MSigDB) (28) (Table S6). Compared to the uninfected BMDMs group, 31 and 29 gene sets were significantly enriched in L. interrogans- and *L. biflexa*-infected BMDMs at 2 h postinfection, respectively, whereas three significantly enriched pathways, “HALLMARK_REACTIVE_OXYGEN_SPECIES_PATHWAY,”“HALLMARK_TGF_BETA_SIGNALING,” and “HALLMARK_GLYCOLYSIS,” were identified in L. interrogans-infected BMDMs but not in *L. biflexa*-infected BMDMs (Fig. 5A–C). At 24 h postinfection, 18 and 16 gene sets were significantly enriched in L. interrogans- and *L. biflexa*-infected BMDMs, respectively, whereas the “HALLMARK_UV_RESPONSE_UP” and “HALLMARK_PI3K_AKT_ MTOR_ SIGNALING” gene sets were only found in L. interrogans-infected BMDMs (Table S6 and Fig. S2). The ranked lists of the core enrichment genes (also known as leading-edge genes) with the highest metric scores for each enriched gene set are shown in Table S7. These core enrichment genes are thought to be actively regulated by pathogenic L. interrogans infection and have the greatest effect on biological processes.

A recent study demonstrated that hypoxia-inducible factor-1α (HIF-1α), a metabolic regulator, promotes macrophage glycolysis metabolism, resulting in proinflammatory macrophage functional differentiation and protection against bacterial and fungal infections (29).

Our RNA-seq transcriptome data showed that HIF-1α was differentially expressed in *Leptospira*-infected BMDMs. Validation using quantitative RT-PCR analysis confirmed that the expression of HIF-1α was significantly upregulated in *L. biflexa*-infected BMDMs compared to in uninfected BMDMs (*P < *0.005 for 2-h infection; *P < *0.05 for 24-h infection) (Fig. 5D and Table S3). The HIF-1α mRNA levels were significantly upregulated in L. interrogans-infected BMDMs compared to in uninfected BMDMs (*P < *0.005) and *L. biflexa*-infected BMDMs (*P < *0.01, 2-h infection; *P < *0.005 for 24-h infection) (Fig. 5D). These results implicate the HIF-1α-dependent glycolysis pathway in the activation of macrophages induced by pathogenic leptospiral infection.

### Attenuating effect on macrophage activation induced by nonpathogenic leptospiral infection.

**FIG 6:**
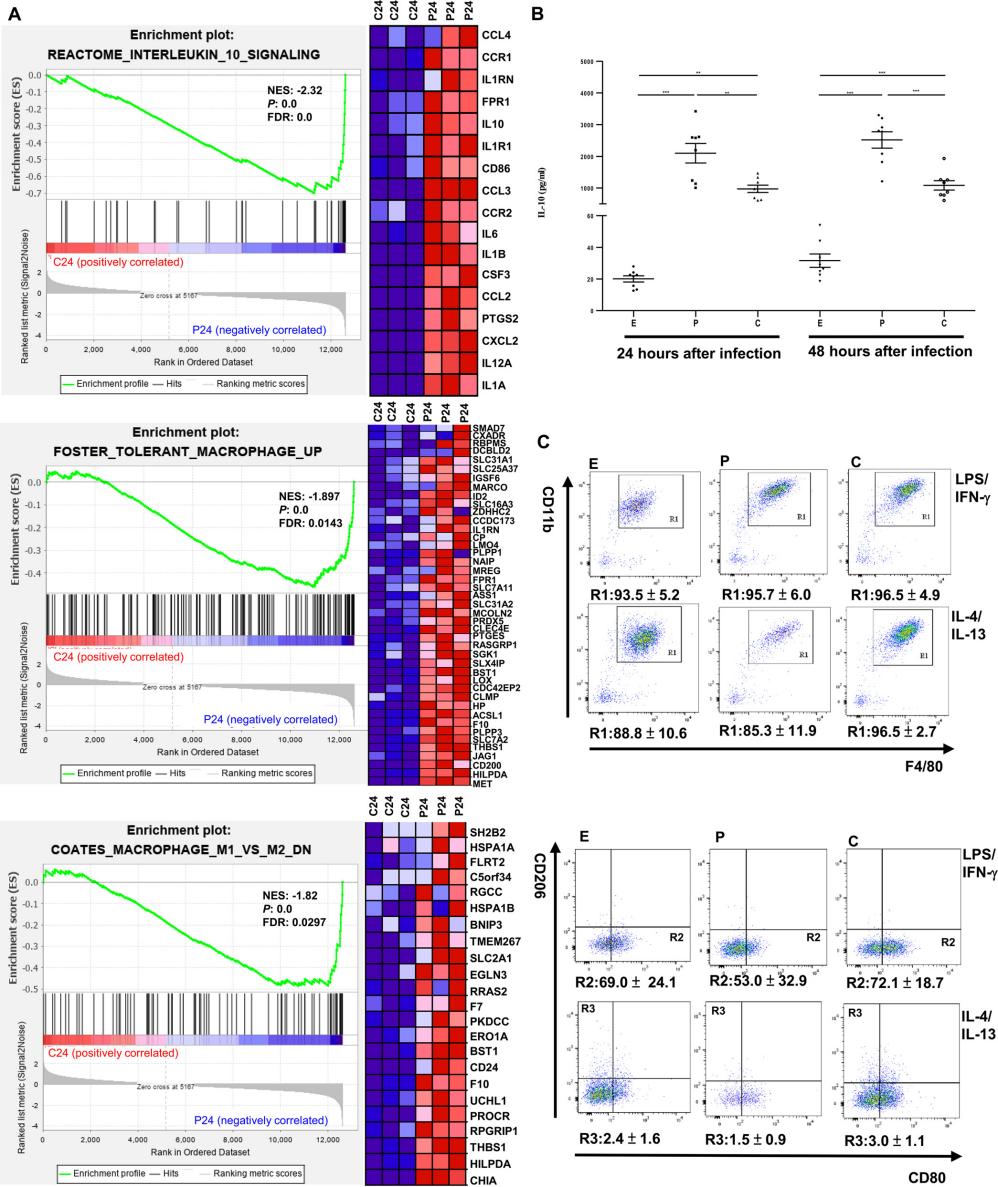
Activated macrophages after infection with nonpathogenic *Leptospira* spp. at 24 h. (A) Plots of gene set enrichment analysis results of “REACTOME_ INTERLEUKIN_10_SIGNALING,” “FOSTER_TOLERANT_MACROPHAGE_UP,” and “COATES_MACROPHAGE_M1_VS_ M2_DN” in *L. biflexa*-infected BMDMs when L. interrogans-infected BMDMs compared to in *L. biflexa*-infected BMDMs. (NES absolute value of >1, *P < *0.05, and FDR < 0.25). The green curve refers to the calculation of the enrichment score. BMDM, bone marrow-derived macrophage; GSEA, gene set enrichment analysis; NES, normalized enrichment score; FDR, false discovery rate. Left panels show GSEA enrichment plots (score curves), and the heatmap on the right side of each panel is a visualization of the core enrichment genes (positively enriched associated molecules) contributing the most to the enriched pathway. Increased expression (red) and decreased expression (blue). C24: L. interrogans-infected BMDMs at 24 h postinfection; P24: *L. biflexa*-infected BMDMs at 24 h postinfection. (B) Effect of IL-10 concentration in the culture supernatant of *Leptospira*-infected BMDMs. E: uninfected BMDMs; P: *L. biflexa*-infected BMDMs; C: L. interrogans-infected BMDMs. The data were analyzed using two-way analysis of variance followed by multiple comparisons tests (GraphPad Prism). Significant differences are representative of the results from multiple experimental replicates (*n *= 8). **, *P < *0.01; ***, *P < *0.005. (C) M1 and M2 macrophage polarization in BMDMs infected with *Leptospira* spp. Representative flow cytometry analysis of BMDMs was defined as CD11b^+^F4/80^+^ cells (gate R1), M1 was defined as CD11b^+^F4/80^+^CD80^+^ subset (gate R2), and M2 was defined as CD11b^+^F4/80^+^CD206^+^ subset (gate R3). Under the representative fluorescence-activated cell sorting plots, the mean proportion of cell subsets is shown, and the data are shown as the mean ± standard error of the mean data from three independent experiments (*n *= 3).

To expand upon the results above, significantly enriched gene sets were identified in L. interrogans-infected BMDMs compared to in *L. biflexa*-infected BMDMs using MSigDB gene sets C2 (curated gene sets containing 4,738 gene sets). When L. interrogans-infected BMDMs were compared with *L. biflexa*-infected BMDMs at 24 h after infection, gene sets with a positive NES acted primarily as functional pathways for activated macrophages induced by pathogenic leptospiral infection, whereas those with a negative NES acted primarily as functional pathways for activated macrophages induced by nonpathogenic leptospiral infection. The results revealed two significantly regulated C2 gene sets in L. interrogans-infected BMDMs: “REACTOME_BRANCHED_CHAIN_AMINO_ACID_CATABOLISM” (NES = 2.15, nominal *P* value = 0.0, FDR q-value = 0.12) and “REACTOME_THE_ROLE_OF_NEF_IN_ HIV_1_REPLICATION_AND_DISEASE_PATHOGENESIS” (NES = 1.89, nominal *P* value = 0.0, FDR q-value = 0.24). We also found significant enrichment of 379 regulated C2 gene sets in *L. biflexa*-infected BMDMs. Among the three biological pathways, “REACTOME_INTERLEUKIN_10_SIGNALING,” “FOSTER_TOLERANT_MACROPHAGE _UP,” and “COATES_MACROPHAGE_M1_VS_ M2_DN” were enriched in *L. biflexa*-infected BMDMs but not in L. interrogans-infected BMDMs, indicating that nonpathogenic leptospiral infection affects macrophage activation (Fig. 6A). In the significantly regulated C2 gene set, “REACTOME_INTERLEUKIN_10_SIGNALING,” the gene encoding IL-10, an anti-inflammatory cytokine, was found in the ranked lists of leading-edge genes containing 17 core enrichment genes, shown as a heatmap for three *L. biflexa*-infected BMDMs and three L. interrogans-infected BMDMs, whereas IL-10 expression was upregulated in *L. biflexa*-infected BMDMs (Fig. 6A). This finding was verified using quantitative RT-PCR analysis, which showed that IL-10 mRNA levels in *L. biflexa*-infected BMDMs were significantly higher than those in cells infected with L. interrogans for 24 h (*P < *0.01) (Fig. 4B). Furthermore, at 24 h (*P < *0.01) and 48 h (*P < *0.005) postinfection, the culture supernatant of *L. biflexa*-infected BMDMs displayed significantly higher IL-10 levels than that of L. interrogans-infected BMDMs (Fig. 6B). Thus, macrophages infected with nonpathogenic *Leptospira* spp. induced higher levels of IL-10 mRNA and protein than those infected with pathogenic *Leptospira* spp.

Because the gene set “COATES_MACROPHAGE_M1_VS_ M2_DN,” which defines a set of downregulated genes differentiating between M1 and M2 macrophage subtypes, was significantly enriched in *L. biflexa*-infected BMDMs, the phenotype and frequency of the polarized macrophages induced by leptospiral infection were assessed using flow cytometry analysis at 48 h postinfection (Fig. 6C). We further evaluated the relative effect of pathogenic and nonpathogenic leptospiral infections on macrophage polarization using *ex vivo* models. As shown in Fig. 6C, under M1 or M2 priming conditions, flow cytometric analysis revealed that a substantial proportion (>85%) of cells were doubly positive for the macrophage markers F4/80 and CD11b, regardless of leptospiral infection. Under the M1 priming condition (infected cells stimulated with LPS/interferon-γ for a second 24-h period), the percentage of the CD80^+^ macrophage subset (gate R2) gated on CD11b^+^F4/80^+^ cells (gate R1) was lower in *L. biflexa*-infected BMDMs (53.0 ± 32.9% of CD11b^+^F4/80^+^CD80^+^ cells, quantitatively normalized to the total number of cells) than in uninfected BMDMs with LPS/interferon-γ stimulation (69.0 ± 24.1% of CD11b^+^F4/80^+^CD80^+^ cells, quantitatively normalized to the total number of cells), whereas there was no obvious difference in the percentage of CD11b^+^F4/80^+^CD80^+^ cells between L. interrogans-infected and uninfected cells. Under the M2 priming condition (infected cells stimulated with IL-4/IL-13 for a second 24-h period), *L. biflexa*-infected BMDMs showed a slightly lower percentage of the CD206^+^ macrophage subset (gate R3) gated on CD11b^+^F4/80^+^ cells (gate R1) than L. interrogans-infected and uninfected BMDMs (full gating strategy is shown in Fig. S3).

Our findings reveal that nonpathogenic *Leptospira*-infected BMDMs treated under M1 or M2 priming conditions inhibited macrophage polarization, particularly macrophage M1 polarization. Taken together, the attenuating effect on macrophage activation is caused by nonpathogenic leptospiral infection.

### Discussion.

We examined overall gene expression alterations and regulation in pathogenic and nonpathogenic *Leptospira*-infected macrophages. Based on their pathogenicity, *Leptospira* species have been categorized into three different clusters: pathogenic, intermediate, and nonpathogenic (saprophytic) *Leptospira* spp., and more than 300 serovars of *Leptospira* have been identified (1, 30). Leptospirosis is caused by pathogenic leptospires, which induce severe, moderate, or asymptomatic infections in humans (2). Pathogenic leptospiral infection causes a vigorous inflammatory response because of its virulence components, such as LPS, peptidoglycans, lipoproteins, glycoproteins, and membrane proteins (2). Nonpathogenic *L. biflexa* is a free-living bacterium found in water and soil but does not infect humans. Previous studies revealed that rabbit antisera produced by inoculation with pathogenic leptospires cross-react with antigenic components derived from the LPS fraction of nonpathogenic leptospires. Membrane components from nonpathogenic leptospires can be used as leptospirosis vaccines. Understanding the effects of pathogenic and nonpathogenic *Leptospira* spp. on hosts is equally important.

Renal leptospirosis, characterized by tubulointerstitial nephritis and tubular dysfunction, is associated with pathogenic *Leptospira* species that chronically infect and harbor bacteria in renal tubules, causing an inflammatory milieu followed by kidney injury and repair. Our previous studies described global changes in renal gene expression during leptospiral infection, and we performed a comprehensive transcriptome analysis of both renal-*Leptospira* species interaction partners using high-throughput NGS (6). Leptospiral genes were detected in the renal transcriptome of mice infected with L. interrogans at day 28 postinfection, suggesting that leptospires colonized the kidney and showed the propensity for chronicity. Comparative pathway analysis showed that TLR signaling, complement activation, T-helper 1 type immune response, and T cell-mediated immunity/chemotaxis/proliferation were strongly associated with progressive tubulointerstitial damage caused by pathogenic leptospiral infection (5, 6). The findings regarding the cytokine and chemokine cascades involved in *Leptospira*-infected kidney tissue may explain the role of the renal microenvironment in kidney damage induced by *Leptospira* spp.

Ultrastructural changes in cellular morphology and bacterial uptake within macrophages infected with nonpathogenic or pathogenic *Leptospira* species were characterized using TEM. Cells infected with *L. biflexa* or L. interrogans exhibit distinct morphological features. We found that intact L. interrogans was localized in the cytoplasm of BMDMs at 2 and 24 h after infection, suggesting that L. interrogans survived within the macrophages (Fig. S1B). Macrophages infected with *L. biflexa* contained no intact bacterial cells in the cytoplasmic vacuole (Fig. S1B, arrowhead), suggesting that leptospires were degraded in nonpathogenic *L. biflexa*-infected BMDMs. Our results are consistent with previous research findings (9). These findings indicate that the fate of intracellular leptospires depends on the differences between pathogens and nonpathogens, which may be associated with macrophage activation and immune evasion. Interestingly, Shetty et al. found that when macrophages fail to clear pathogenic L. interrogans, a chemokine/cytokine storm occurs, resulting in a robust but nonprotective inflammatory response to pathogenic *Leptospira* spp., leading to infection spread, kidney colonization, and pathological effects (31).

Macrophages sense microorganisms *via* PRRs and trigger intracellular signaling cascades, resulting in transcriptional expression of inflammatory and anti-inflammatory cytokines. Macrophage PRRs, including TLRs, NLRs, C-type lectin receptors, mannose, and scavenger receptors, recognize and bind to microbial products to initiate phagocytosis (8). In the KEGG and IPA pathway enrichment for *Leptospira*-infected BMDMs after the initial infection (at 2 h), PRR-signaling pathways including the “C-type lectin receptor signaling pathway,” “NOD-like receptor signaling pathway,” and “TLR signaling pathway” were identified in nonpathogenic *Leptospira*-infected BMDMs, whereas the “NOD-like receptor signaling pathway” and “TLR signaling pathway” were found in pathogenic *Leptospira*-infected BMDMs. Activation of NOD signaling may be associated with enhanced cytokine production and induction of inflammatory and antimicrobial responses to protect against infection (32). L. interrogans escapes NOD recognition through peptidoglycan modification induced by leptospiral lipoprotein LipL21 binding (7). According to our findings, the DEGs were significantly enriched in pathways associated with neutrophil extracellular trap formation in pathogenic *Leptospira*-infected BMDMs at 24 h after infection. Doster et al. (33) reported that neutrophil extracellular traps have features similar to those of macrophage extracellular traps, suggesting that macrophage extracellular traps are involved in leptospiral infection mechanisms or play a role in tissue diseases.

Among the significantly enriched IPA canonical pathways, TREM1 and IL-10 signaling were enriched in both L. interrogans- and *L. biflexa*-infected BMDMs, regardless of the infection period. TREM1 activation in macrophages triggers signaling pathways that positively modulate proinflammatory responses, augmenting the secretion of proinflammatory chemokines and cytokines in response to infections (34). Most importantly, the anti-inflammatory cytokine IL-10 is a key immunoregulator during infection and is induced in macrophages *in vitro* when TLR2 signaling is modulated during Candida albicans infection or after exposure to the human pathogenic *Yersinia* spp. virulence V protein (35). Macrophage-derived IL-10 suppresses macrophage development into classically activated macrophages, inhibits apoptosis of Mycobacterium avium-infected cells, and leads to the induction of CD4^+^CD25^+^ regulatory T cells (35). We found that pathogenic *Leptospira*-infected BMDMs had higher TREM1 transcript expression compared to that in nonpathogenic *Leptospira*-infected BMDMs, whereas nonpathogenic *Leptospira*-infected BMDMs showed higher IL-10 transcript expression compared to that in pathogenic *Leptospira*-infected BMDMs. We predict that nonpathogenic *Leptospira*-infected macrophages produced the anti-inflammatory cytokine IL-10, which may be involved in polarizing macrophages into an anti-inflammatory activation state.

We found that CCL2, C-C motif chemokine ligand 7 (CCL7), C-C motif chemokine ligand 3 (CCL3), CXCL1, C-X-C motif chemokine ligand 2, C-X-C motif chemokine ligand 3, C-X-C motif chemokine ligand 5, IL-6, IL-19, IL-27, and IL-33 gene transcript levels were higher in nonpathogenic *Leptospira*-infected BMDMs than in pathogenic *Leptospira*-infected BMDMs at 24 h after infection (Table S4). Both CCL2 and IL-6 prevent apoptosis to promote CD11b^+^ cell survival and induce M2 type macrophage activation (36). Furthermore, according to the literature, IL-6 and IL-27 are classified as proinflammatory or anti-inflammatory factors in acute and chronic inflammation models (37). IL-27 decreases proinflammatory cytokines and T cell subsets directly or exert anti-inflammatory effects in macrophages by promoting increased expression of IL-10. Previous studies highlighted that CCL7 plays a negative regulatory role in restricting inflammation following Leishmania major infection and the ability of the parasite to disseminate (38). CCL3 may play an important role in phagocyte activation during antibacterial host defense by inducing proinflammatory cytokines, which can help phagocytes destroy intracellular pathogens more effectively (39). Furthermore, IL-19 is upregulated in macrophages after infection and reduces inflammation (40). A previous study showed that IL-33 is important for host resistance because it favors the M2 phenotype, stimulates IL-10 synthesis, and inhibits proinflammatory cytokine expression (41).

At 24 h after leptospiral infection, pathogenic *Leptospira*-infected BMDMs with low IL-10 expression had higher C-C motif chemokine ligand 8 (CCL8), C-C motif chemokine receptor 3 (CCR3), CXCL10, CXCL9, C-C motif chemokine ligand 5 (CCL5), monocyte chemotactic protein-5, and complement C3 (C3) gene transcript expression than nonpathogenic *Leptospira*-infected BMDMs with high expression of IL-10 (Table S5). Liu et al. reported that Mycobacterium tuberculosis stimulated CCL8 synthesis in macrophages at both the transcriptional and protein levels, depending on activation of the TLR2/PI3K/Akt and p38 signaling pathways (42). The “HALLMARK_PI3K_AKT_MTOR_SIGNALING” gene sets were enriched in L. interrogans-infected BMDMs but not in *L. biflexa*-infected BMDMs (Fig. S2). CCL8 also interacts with CCR3 (43). A previous study confirmed that the chemokines expressed during both acute and chronic infections with the protozoan parasite Trypanosoma cruzi are CXCL9, CXCL10, and CCL5 produced by macrophages, indicating a protective host response to T. cruzi infection (44). Complement systems, such as C3, are involved in infection, with local C3 secretion by macrophages promoting renal fibrosis and defense mechanisms against intracellular bacteria (45).

In conclusion, the IL-10 signature has a biological importance in activated macrophages infected with leptopsires. Nonpathogenic *Leptospira*-infected macrophages with high IL-10 production may play a negative regulatory role in macrophage activation and an attenuated response in classical macrophage activation. Our results showed that immune-related biological processes were significantly activated in BMDMs infected with pathogenic *Leptospira* spp. at 2 and 24 h postinfection. After infection for 2 h, the HIF-1α-dependent glycolysis pathway may be involved in activated macrophages induced by pathogenic leptospiral infection. Classically activated macrophages are the major polarization status of pathogenic *Leptospira*-infected macrophages. Therefore, macrophages infected with pathogenic *Leptospira* spp. may create inflammatory microenvironments by balancing the expression of anti-inflammatory molecules, such as IL-10, resulting in chronic or persistent tissue damage. Our findings must be confirmed in animal experiments or clinical human studies. This study improves the understanding of transcriptional regulation during infection and provides insight into the molecular pathogenic mechanisms underlying macrophage infection with *Leptospira* spp.

## MATERIALS AND METHODS

### *Leptospira* strains and culture conditions.

L. interrogans serovar Copenhageni Fiocruz L1-130 (pathogenic species) and *L. biflexa* serovar Patoc (nonpathogenic species) were cultivated at 28°C under aerobic conditions in liquid Elinghausen-McCullough-Johnson-Harris medium (Difco, Detroit, MI, USA) for passaging. Prior to infection experiments, L. interrogans was inoculated intraperitoneally into Golden Syrian hamsters (4 weeks old) and recovered from the kidneys of infected hamsters to maintain their virulence (46). For *in vitro* experimental infection, bacterial strains that had been passaged fewer than eight times were used. After 7 days of culture, motile leptospires was counted using dark-field microscopy and a Petroff-Hausser counting chamber for experimental infections. Animal biosafety level 2 conditions were used for all animal experiments, and the experimental protocols were approved by the Institutional Animal Care and Use Committee of the Chang Gung Memorial Hospital in Taiwan (certificate no. 2018062701).

### Differentiation of murine BMDMs.

Femurs and tibias were obtained from 6–10-week-old C57BL/6 mice, and their muscles were removed using protocols approved by the Institutional Animal Care and Use Committee of Chang Gung Memorial Hospital (permit number 2018121710). Both ends of the bones were cut with scissors, and bone marrow cells were extruded with Dulbecco’s modified Eagle medium/F12 (GlutaMAX supplement, Gibco, Grand Island, NY, USA) supplemented with 10% fetal bovine serum (Life Technologies, Carlsbad, CA, USA) using a 25-gauge needle. Bone marrow cells were cultured in complete macrophage medium supplemented with recombinant macrophage colony-stimulating factor (ProSpec-Tany TechnoGene Ltd., Rehovot, Israel). After 7 days of culture, nonadherent cells were eliminated, and adherent cells were harvested for assays. Phenotypic characterization of BMDMs identified as F4/80^+^/CD11b^+^/CD11c- was assayed using flow cytometry (BD FACSVerse; BD Biosciences, Franklin Lakes, NJ, USA) (47). Macrophages were incubated with rat anti-mouse CD16/CD32 (BD Biosciences) to block the Fc receptors. The cells were then stained using the following anti-mouse antibodies: CD11b-APC (M1/70, BioLegend, San Diego, USA), F4/80-Brilliant Violet 420 (BM8, BioLegend), and CD11c-Alexa Fluor 488 (N-418, STEMCELL Technologies, Vancouver, Canada). Data were analyzed using FlowJo software (version 8.8.6, TreeStar, Ashland, OR).

### Ex vivo infection models of *Leptospira* spp.

*Leptospires* were harvested by centrifugation at 4,000 × *g* for 30 min and washed three times with sterilized PBS. BMDMs were seeded at a density of 1 × 10^6^ cells per well in 6-well plates and incubated with pathogenic and nonpathogenic *Leptospira* spp. in fresh complete macrophage medium without antibiotics for 2 or 24 h at 37°C and 5% CO_2_. The multiplicity of infection was 100 bacteria per cell. For RNA extraction, cell samples were collected at 2 and 24 h after infection. The efficiency of leptospiral infection was examined using confocal microscopy (SP8, Leica, Wetzlar, Germany). Apoptotic cell death caused by leptospiral infection was measured using the fluorescein isothiocyanate (FITC) annexin V-propidium iodide (PI)-based method via flow cytometry to differentiate cells in early apoptosis (annexin V+/PI-) from those in post-apoptosis/necrosis (annexin V+/PI+). Data were analyzed using FlowJo software (version 8.8.6). To obtain fully polarized classical (M1) activated macrophages, at 24 h after leptospiral infection, infected BMDMs were stimulated with 10 ng/mL LPS (Sigma-Aldrich, St. Louis, MO, USA) and mouse IFN-γ at 100 ng/mL (BioLegend) for 24 h. For fully polarized alternative (M2) activated macrophages, at 24 h after leptospiral infection, infected BMDMs were stimulated with mouse IL-4 (20 ng/mL, BioLegend) and mouse IL-13 (20 ng/mL Biolegend) for 24 h (18). The cells were harvested and incubated with Fc blocking antibodies. The fraction of cells in each gate that originated from either the M1 or M2 priming conditions was calculated based on cells stained with a phycoerythrin/cyanine 7 (PE/CY7)-conjugated monoclonal antibodies to CD80 (M1 markers, 16-10A1, BioLegend) and FITC-conjugated monoclonal antibodies to CD206 (M2 markers, C068C2, BioLegend) (48). The data are shown as the mean of triplicates ± standard deviation.

### Total RNA extraction.

Total RNA was extracted from cells using TRIzol reagent (Invitrogen, Carlsbad, CA, USA), followed by treatment with RNase-free DNase I according to the manufacturer’s protocol. The quantity and quality of extracted RNA were verified using a NanoDrop spectrophotometer (Thermo Fisher Scientific, Waltham, MA, USA) and Agilent 2100 Bioanalyzer (Agilent Technologies, Santa Clara, CA, USA).

### High-throughput NGS and bioinformatics analysis.

All procedures were performed according to the Illumina protocol (Illumina, San Diego, CA, USA). Amplified cDNA was prepared from total RNA, and RNA-seq library construction was performed using the Ovation RNA-seq System V2 and Ovation Ultralow Library System V2 (NuGEN Technologies, San Carlos, CA, USA), respectively, following standard protocols. The concentration and quality of the library were quantified using Qubit (Invitrogen) and Agilent 2100 Bioanalyzer, and paired-end 150 bp sequencing was carried out on the Illumina HiSeq 4000 sequencing platform. The raw data were filtered using the CLC Genomics Workbench version 10.1, based on a per-base sequence quality score (Q) of ≥20. To quantify transcript expression, sequencing data were mapped to the Mus musculus GRCm38 reference genome. For genome mapping, the length of reads mapped to mouse and similarity fraction of the alignment was set to 80%. PCA was performed using Partek Genomics Suite software (Partek, St. Louis, MO, USA) to assess the similarity of genomic-specific gene expression patterns among different groups. The number of reads aligned to each genomic position was calculated, and the reads per kilobase of exon per million reads method was used to calculate gene expression levels. Up- and downregulated DEGs showing a fold change ≥2 or ≤–2, as well as *P* values of <0.05, were considered as significant and subjected to further bioinformatic analysis. Venn diagram analysis was performed using an Internet tool (http://bioinformatics.psb.ugent.be/webtools/Venn/). For functional analysis, the DEGs were subjected to KEGG pathway enrichment analyses, and *P* values less than 1E-5 were considered to indicate significant enrichment by the DEGs using ClusterProfiler (49) and IPA Build 2021-02-18 (version 62089861). GSEA, which associates gene sets and phenotypes, was performed using GSEA software and gene sets from MSigDB (28, 50). Gene sets with a nominal *P* value of <0.05 and an FDR q-value of <0.25 (25%) were identified as significant. Significantly enriched pathways were displayed using a GSEA plot.

### Immunofluorescence and electron microscopy.

A total of 1 × 10^6^ BMDMs in a volume of 2 mL was seeded onto coverslips in 6-well plates and allowed to adhere. The cell monolayer was infected with pathogenic and nonpathogenic *Leptospira* spp. at a multiplicity of infection of 100 in fresh complete macrophage medium without antibiotics. For the immunofluorescence assay, uninfected and infected cells were fixed in 4% paraformaldehyde, permeabilized with 0.2% Triton X-100, and blocked to prevent nonspecific staining. The cells were then incubated overnight at 4°C with the primary antibody: an anti-Lipl32 antibody to L. interrogans and anti-*L. biflexa* antibody (ab20110; Abcam, Cambridge, UK) to *L. biflexa*. The cells were washed and incubated with an Alexa Fluor 488-conjugated secondary antibody (Molecular Probes, Eugene, OR) for 1 h, followed by nuclear staining with 4′,6-diamidino-2-phenylindole (DAPI; Thermo Fisher Scientific). The cells were photographed using a Leica TCS SP8 X confocal microscope (Leica Microsystems). To adjust brightness and contrast, as well as generate overlay pictures, Leica Application Suite Core X software (version 2.0) was used, and the fluorescence intensity in the images acquired in the same batch with identical optical setup and parameters was quantified. The morphology of the infected macrophages and presence of *Leptospira* spp. in the cells were visualized using TEM. The cells were fixed in 3% glutaraldehyde and 2% paraformaldehyde in cacodylate buffer (0.1 M, pH 7.4), postfixed in 1% osmium tetroxide, and dehydrated with a graded ethanol series (30%, 50%, 70%, 90%, 95%, and 100%). The samples were embedded in epoxy resin and polymerized. Ultrathin sections (80 nm) were obtained from the resin blocks. The sections were double-stained with uranyl acetate (4%) and lead citrate, then loaded onto an electron microscopy grid and observed using an ultra-high-resolution TEM (Hitachi HT7800, Tokyo, Japan) at 100 kV and a magnification of 5,000–27,000× to detect ultrastructural changes.

### Validation using quantitative RT-PCR.

To validate the DEGs, total RNA was extracted as described above, and quantitative RT-PCR was performed using a first-strand cDNA synthesis kit for RT-PCR (Roche Diagnostics, Basel, Switzerland) and an ABI ViiA7 real-time PCR system (Applied Biosystems, Foster City, CA) using TaqMan gene expression assays. The results are expressed as a threshold cycle (*Ct*). The expression level of each gene was normalized to that of GAPDH, and the relative mRNA expression data were calculated using the 2^-ΔΔ^*^Ct^* method (51). All experimental samples were compared with the control (uninfected BMDM groups) and expressed as an *n*-fold difference. The RT-PCR data were statistically analyzed by determining the fold change values. The primer sequences are listed in Table S3.

### Quantification of cytokine levels.

Supernatants from infected BMDMs were harvested and stored at −80°C until analysis. IL-10 levels were measured using commercial ELISA kits (IL-10: Quantikine, R&D Systems, Minneapolis, MN) in accordance with the manufacturer's instructions. Absorbance was measured at 450 nm using an ELISA reader (PowerWave XS; Bio-Tek Instruments, Winooski, VT).

### Statistical analysis.

GraphPad Prism 5.0 (GraphPad Software, Inc., La Jolla, CA) was used for all statistical analyses. Differences between the treatment groups were determined using a two-way analysis of variance followed by multiple comparison tests. Differences were considered as significant when *P* was <0.05. For high-throughput NGS data analysis, raw sequencing data quality control and mapping statistical analysis were performed using CLC Genomics Workbench.

### Data availability.

The RNA sequencing data have been deposited in the Gene Expression Omnibus under accession number GSE166050.
