# Supplementary material for: Implication of the IL-10-Expression Signature in the Pathogenicity of Leptospira-Infected Macrophages
Source: Microbiol Spectr. 2022 May 31;10(3):e02595-21. doi: 10.1128/spectrum.02595-21 (PMC9241676; doi:10.1128/spectrum.02595-21)
Supplement: SUPPLEMENTAL FILE 1 — Supplemental material. Download spectrum.02595-21-s001.pdf, PDF file, 0.6 MB [file spectrum.02595-21-s001.pdf]

Supplemental Material

Implication of the IL-10-expression signature in the pathogenicity of *Leptospira*-infected macrophages

Li-Fang Chou,[a] Ting-Wen Chen,[b,c,d] Huang-Yu Yang,[a,e,f] Ya-Chung Tian,[a,e,f] Ming-Yang Chang,[a,e,f] Cheng-Chieh Hung,[a,e,f] Chih-Ho Lai,[g,h] Shen-Hsing Hsu,[a] Chung-Ying Tsai,[a] Yi-Ching Ko,[a] Jang-Hau Lian,[i] and Chih-Wei Yang [a,e,f #]

[a]Kidney Research Center, Chang Gung Memorial Hospital, Linkou, Taiwan

[b]Institute of Bioinformatics and Systems Biology, National Yang Ming Chiao Tung University, Hsinchu, Taiwan

[c]Department of Biological Science and Technology, National Yang Ming Chiao Tung University, Hsinchu, Taiwan

[d]Center for Intelligent Drug Systems and Smart Bio-devices (IDS^{2}B), National Yang Ming Chiao Tung University, Hsinchu, Taiwan

[e]Department of Nephrology, Chang Gung Memorial Hospital, Linkou, Taiwan

[f]College of Medicine, Chang Gung University, Taoyuan, Taiwan

[g]Department of Microbiology and Immunology, Chang Gung University, Taoyuan, Taiwan;

[h]Molecular Infectious Disease Research Center, Chang Gung Memorial Hospital, Taoyuan, Taiwan;

[i]Genomic Medicine Core Laboratory, Chang Gung Memorial Hospital, Linkou, Taiwan

Running Head: IL-10 expression in *Leptospiral*-infected macrophages

#Correspondence: Prof. Chih-Wei Yang; E-mail: cwyang@ms1.hinet.net; Kidney Research Center and Department of Nephrology, Chang Gung Memorial Hospital, Chang Gung University College of Medicine, 5 Fu-Shing St., Kueishan, Taoyuan, 333, Taiwan. Phone: +886-3-3281200 EXT.7756; Fax: +886-3-3282173

This article contains the following supplemental material

FIG S1. Biological characterization of *Leptospira*-infected bone marrow-derived macrophages (BMDMs). (A) Changes in the density and shape of mitochondria in *Leptospira*-infected BMDMs. Representative transmission electron microscopy images of BMDMs infected with *L. biflexa* (left) or *L. interrogans* (right) for 2 h. Magnification: (upper row) 27,000x, scale bar: 2.0 μm; (lower row) 110,000x, scale bar: 1.0 μm, area delineated by a rectangle in the top row. Black arrow and M indicate mitochondria; N-Nucleus; LBP-*L. biflexa*; LIC- *L. interrogans*. (B) Transmission electron microscopy images of intracellular leptospiral localization patterns in infected macrophages. Images show *L. biflexa*-infected BMDMs at 2 h (upper row) and (lower row) 24 h post-infection (scale bar, 1.0 μm), and *L. interrogans*-infected BMDMs at 2 h (upper low) and (lower row) 24 h post-infection (scale bar, 500 nm and 1.0 μm, respectively). Black arrowheads show *L. biflexa*, arrows show *L. interrogans*, and M* and V indicate damaged mitochondria and vacuoles, respectively. (C) Amount of *L. biflexa* in infected BMDMs was larger than the amount of *L. interrogans*. Quantification of the total fluorescent area (upper row) and fluorescence intensity (lower row) in an infected cell population at the single-cell level. An algorithm for measuring the total fluorescent area and assessing the total cell number on an image was developed in Python 3.10.0 using OpenCV (Open-Source Computer Vision, Intel Corp.). The total fluorescence intensity was measured using MetaMorph software. Cells within 4 images (approximately 80 cells) were analyzed. P: *L. biflexa*-infected BMDMs, and C: *L. interrogans*-infected BMDMs. BMDM, bone marrow-derived macrophage. (D) Induction of apoptosis in BMDMs infected with *Leptospira* spp. After infection with leptospires for (upper row) 2 h and (lower row) 24 h, the percentage of apoptotic cells was detected using a fluorescein isothiocyanate (FITC)-annexin V/propidium iodide (PI) double staining method and analyzed using flow cytometry. Non-apoptotic cells (lower left quadrant); early apoptotic cells (lower right quadrant); late apoptotic/necrotic cells (upper right quadrant).

FIG S2. Macrophage responses after 24 h of infection with pathogenic *Leptospira* spp. Plots obtained from gene set enrichment analysis of "HALLMARK_UV_RESPONSE_UP" and "HALLMARK_PI3K_AKT_MTOR_ SIGNALING" are shown in *L. interrogans*-infected BMDMs *vs.* uninfected BMDM groups but not in *L. biflexa*-infected BMDMs *vs.* uninfected BMDM groups. The green curve refers to calculation of the enrichment score. GSEA, gene set enrichment analysis; NES, normalized enrichment score; FDR, false discovery rate. Left panels show GSEA enrichment plots (score curves) and the heatmap on the right side of each panel shows the core enrichment genes (positively enriched associated molecules) contributing the most to the enriched pathway. Increased expression (red) and decreased expression (blue). C24: *L. interrogans*-infected BMDMs at 24 h post-infection and E24: uninfected BMDMs at 24 h. BMDM, bone marrow-derived macrophage.

FIG S3. Representative flow cytometry plot showing the gating strategy to identify classically activated M1 or alternatively activated M2 macrophage subsets in leptospiral-infected BMDMs. The cells were first morphologically gated using the forward light scatter area (FSC-A) and side light scatter area (SSC-A) to exclude cell debris. Viability gating was performed to select cells that were negative for the eBioscience fixable viability dye stain (Thermo Fisher Scientific, 65-0865-14), and cell doublets were excluded based on the FSC-A and FSH-height (FSH-H). Live cells were gated on $CD11b^+F4/80^+$ cells (R1). Next, $CD11b^+F4/80^+$ cells were analyzed for the expression of CD80 and CD206: R2: $CD11b^+F4/80^+CD80^+$ (classically activated M1) and R3: $CD11b^+F4/80^+CD260^+$ (alternatively activated M2).

Tables and their legends

Table S1. Summary of sequencing data and alignment statistics

Table S2. Enriched canonical pathways of DEGs using Ingenuity Pathway Analysis. The significance of canonical pathways was determined using a default threshold of *p*-values < 1E-5.

Table S3. Candidate genes for Taqman probe ID.

Table S4. At 24 h after infection, pathogenic *Leptospira*-infected macrophages showed lower expression levels of gene transcripts compared to those in nonpathogenic *Leptospira*-infected macrophages.

Table S5. At 24 h after infection, pathogenic *Leptospira*-infected macrophages showed higher expression levels of gene transcripts compared to those in nonpathogenic *Leptospira*-infected macrophages.

Table S6. Pathways significantly enriched in Gene Set Enrichment Analysis.

Table S7. Core enrichment genes

FIG S1.

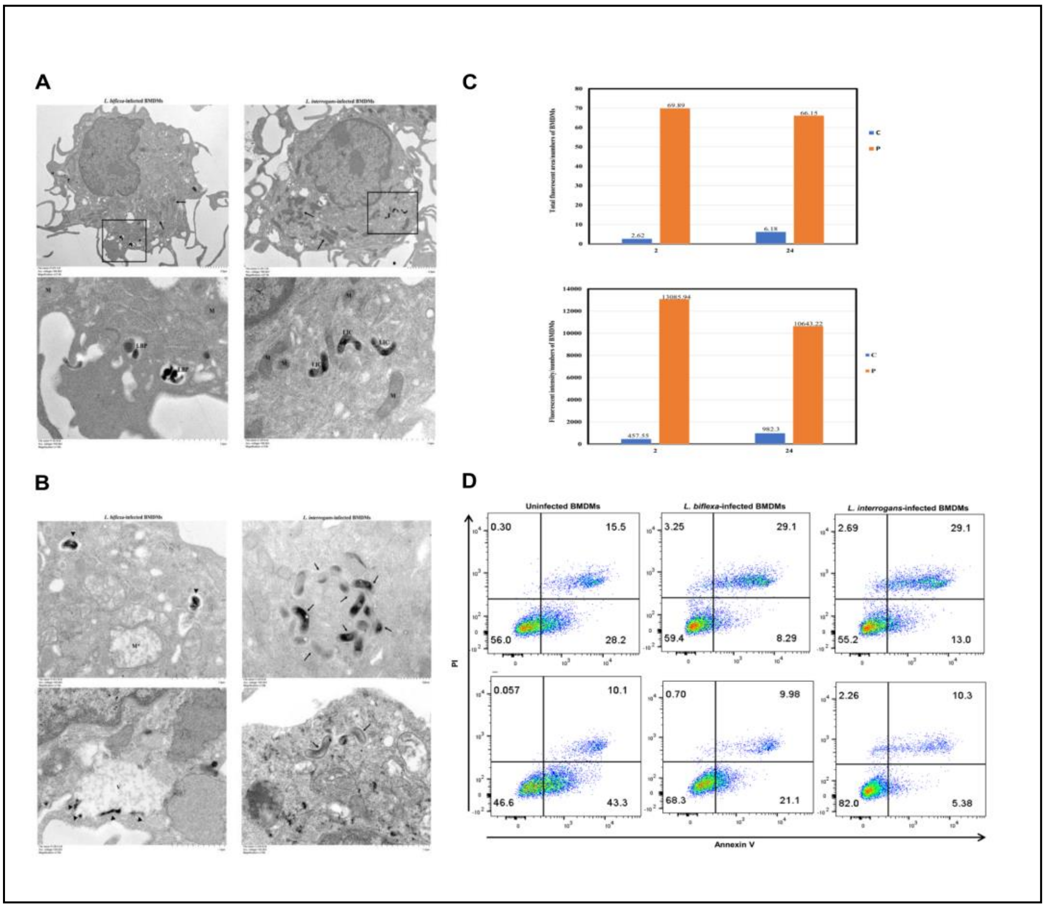

A
B
C
D
69.89
66.15
2.62
6.18
12085.94
10643.22
457.55
982.3
Uninfected BMDMs
L. biflexa-infected BMDMs
L. interrogans-infected BMDMs
0.30
15.5
56.0
28.2
3.25
29.1
59.4
8.29
2.69
29.1
55.2
13.0
0.057
10.1
46.6
43.3
0.70
9.98
68.3
21.1
2.26
10.3
82.0
5.38
PI
Annexin V

FIG S2.

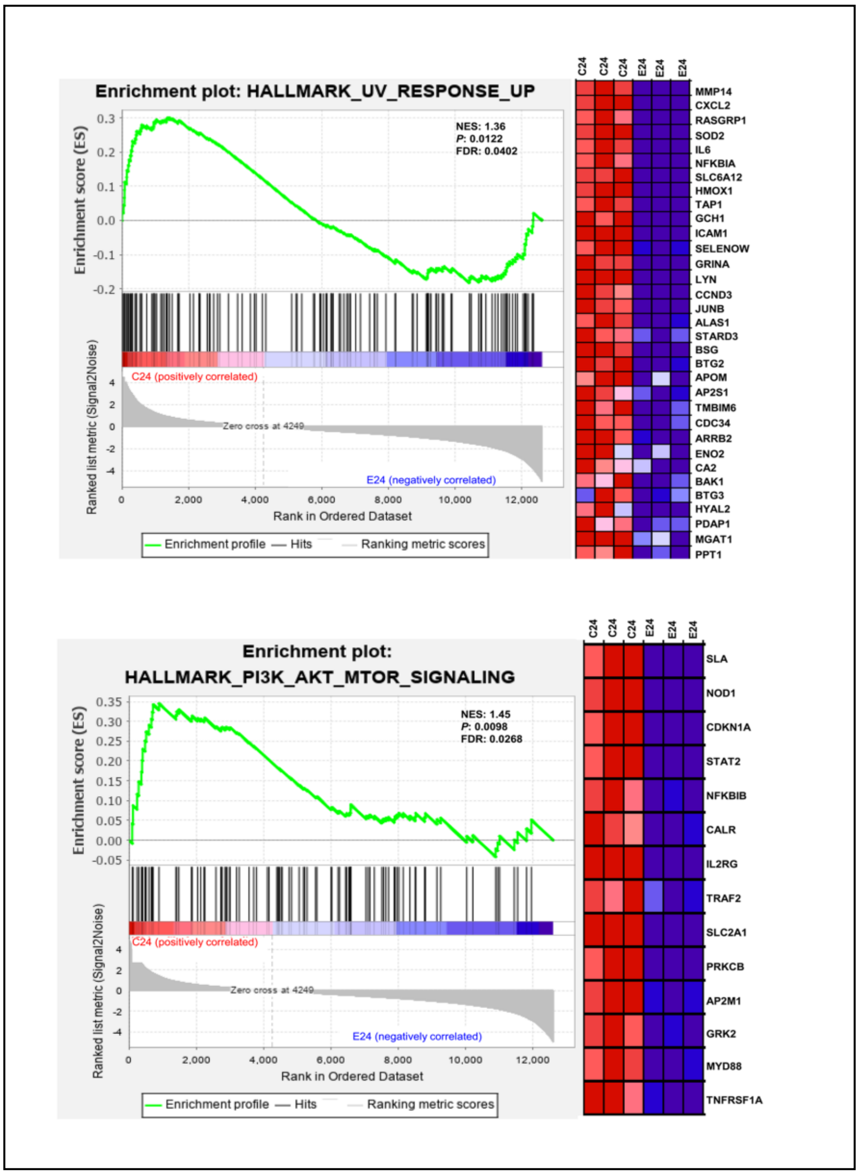

Enrichment plot: HALLMARK_UV_RESPONSE_UP
NES: 1.36
P: 0.0122
FDR: 0.0402
Enrichment score (ES)
Ranked list metric (Signal2Noise)
C24 (positively correlated)
Zero cross at 4249
E24 (negatively correlated)
Rank in Ordered Dataset
Enrichment profile
Hits
Ranking metric scores
C24
E24
MMP14
CXCL2
RASGRP1
SOD2
IL6
NFKBIA
SLC6A12
HMOX1
TAP1
GCH1
ICAM1
SELENOW
GRINA
LYN
CCND3
JUNB
ALAS1
STARD3
BSG
BTG2
APOM
AP2S1
TMBIM6
CDC34
ARRB2
ENO2
CA2
BAK1
BTG3
HYAL2
PDAP1
MGAT1
PPT1
Enrichment plot:
HALLMARK_PI3K_AKT_MTOR_SIGNALING
NES: 1.45
P: 0.0098
FDR: 0.0268
SLA
NOD1
CDKN1A
STAT2
NFKBIB
CALR
IL2RG
TRAF2
SLC2A1
PRKCB
AP2M1
GRK2
MYD88
TNFRSF1A

FIG S3.

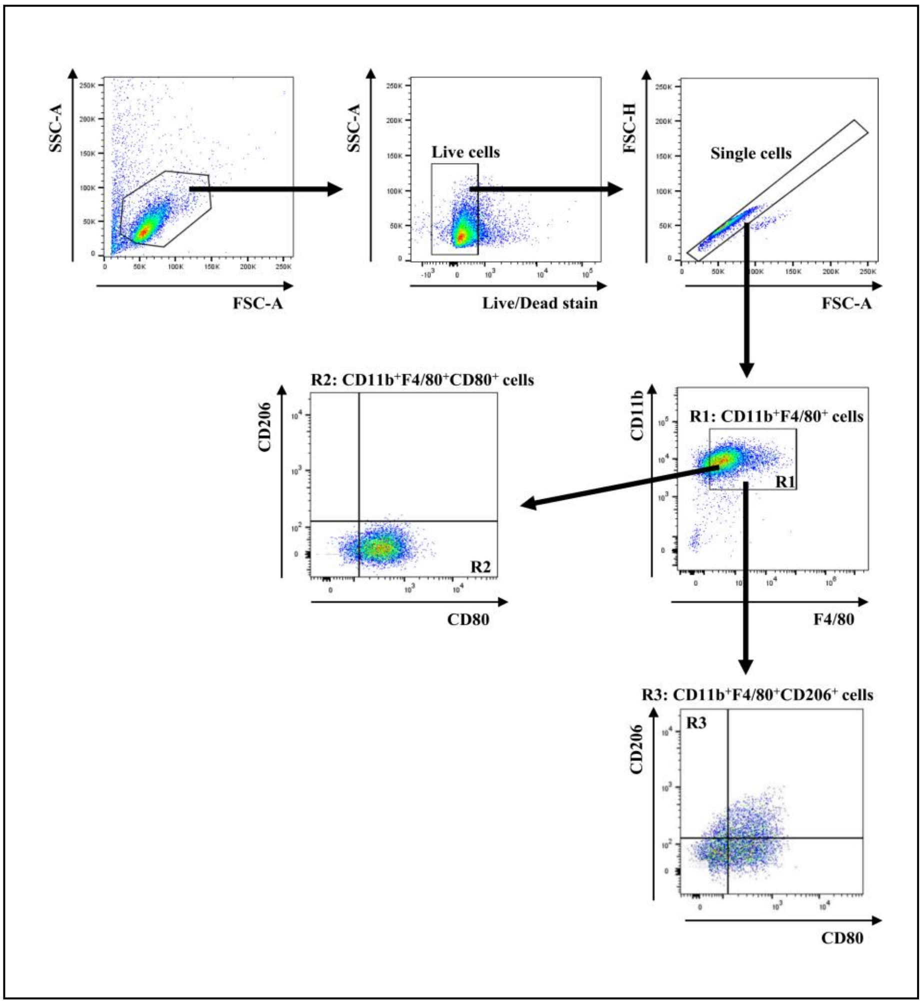
SSC-A
FSC-A
SSC-A
Live cells
Live/Dead stain
FSC-H
Single cells
FSC-A
R2: CD11b+F4/80+CD80+ cells
CD206
R2
CD80
CD11b
R1: CD11b+F4/80+ cells
R1
F4/80
R3: CD11b+F4/80+CD206+ cells
CD206
R3
CD80

Table S1. Summary of sequencing data and alignment statistics

Groups	Infection times[a]	Samples	Total number of clean reads[b]	Total number of clean reads[c]	Total number of mapped reads[d]	Total mapping ratio (%)[e]	Total gene number
**Uninfected BMDMs**	2	1	60.81	60,792,594	59,212,335	97.40	24,495
		2	57.26	57,241,093	55,611,226	97.15	24,468
		3	65.97	65,943,574	64,229,828	97.40	24,661
	24	1	62.26	62,255,382	60,667,230	97.45	32,874
		2	60.39	60,388,092	58,725,491	97.25	33,194
		3	79.18	79,172,384	77,212,919	97.53	34,304
***L. biflexa*-infected BMDMs**	2	1	60.58	60,563,231	59,205,948	97.76	23,406
		2	61.90	61,876,066	60,205,229	97.30	23,725
		3	63.74	63,706,692	62,162,068	97.58	23,411
	24	1	70.52	70,520,481	68,275,316	96.82	34,588
		2	81.37	81,363,336	78,895,826	96.97	33,625
		3	62.55	62,547,567	60,181,836	96.22	32,388
***L. interrogans*-infected BMDMs**	2	1	55.45	55,429,633	54,046,002	97.50	22,984
		2	59.81	59,785,235	58,571,055	97.97	22,919
		3	55.90	55,877,445	54,412,993	97.38	23,238
	24	1	47.95	47,951,630	46,287,713	96.53	32,695
		2	57.20	57,192,970	55,195,931	96.51	31,560
		3	53.23	53,231,811	51,516,773	96.78	31,140

[a] Infection times, hours

[b] Total number of raw reads: The reads amount before filtering, Unit: Mb

[c] Total number of clean reads: The number of clean reads after filtering; removal of low quality sequence (Quality value≧20; sequencing reads trimming and quality control using CLC Genomics Workbench v10.1 (CLC Bio, Denmark)

[d] Total clean reads alignment and mapping on to the reference genome (*Mus musculus* GRCm38)

[e] Total mapping ratio (%): The percentage of mapped reads

Table S3. List of candidate genes for Taqman probe ID.

Gene symbol	Gene name	TaqMan assay ID
NLRP3	NLR family, pyrin domain containing 3	Mm00840904_m1
TLR1	Toll-like receptor 1	Mm00446095_m1
TLR2	Toll-like receptor 2	Mm00442346_m1
TNF-a	Tumor necrosis factor-alpha	Mm99999068_m1
IL-10	Interleukin 10	Mm00439614_m1
CCL2	Chemokine (C-C motif) ligand 2; MCP-1	Mm00441242_m1
IL-6	Interleukin 6	Mm01210733_m1
CXCL1	Chemokine (C-X-C motif) ligand 1	Mm04207460_m1
CCR1	Chemokine (C-C motif) receptor 1	Mm00438260_s1
Emr1	EGF-like module containing, mucin-like, hormone; F4/80	Mm00802529_m1
HIF-1a	Hypoxia inducible factor 1, alpha subunit	Mm01283761_g1
GAPDH	Glyceraldehyde-3-phosphate dehydrogenase	Mm99999915_g1
